# Running the Risk

**Published:** 1872-03

**Authors:** H. L. Sage


### Communications.

### RUNNING THE RISK.

BY. H. L. SAGE.

Running the risk, though, perhaps, justifiable in one's own case, where nothing immoral is involved, is not always, or generally so, where the interests and well-being of others are so concerned in our actions, as to render us wholly, or partially responsible for the results. If, when in matters affecting only the pecuniary interests of others, one assumes responsibility, and is false to his trusts, he merits condemnation, how much more does he, who, in assuming the care of organs, upon whose healthful condition so much depends, is satisfied with the carrying out of make-shift expedients whose good results, if any there are—are only temporary, and which are followed in the not distant future, by permanently bad effects, loss, or ruin.

Running the risk in dental operations, is not uncommon. Sometimes necessary and justifiable, it is, at other times, uncalled for, dangerous and reprehensible. The right and wrong of the matter, is inseparably connected with the expediency and probable results.

First, when is it right? When it is necessary in order to shun a positive, sure, or greater evil, which would otherwise result. When the evils consequent upon running the risk of a certain course of practice are temporary, or less than the present or prospective evils to be avoided.

Running the risk, always implies a doubt—an uncertainty of the consequences.

In many things, we must breast the uncertainty. But in balancing the results of the past experiences of ourselves, and others, in the modes of practice adopted in certain, similar, specified cases, with the existing conditions of the one in hand, we shall be enabled to go forward understandingly.

Certainties and uncertainties should be looked in the face. A mode of procedure uncertain in its results, should not, as a rule, be substituted for one certain to bring about as good or better ones, even though the latter, may require a more tedious and round about course. If the ultimate greater good resulting from a given course outweigh the lesser evil, necessarily tolerated to bring it about, there should be no hesitation as to the carrying out of the latter when the probable result is apparent.

But where serious doubts exist, they should not be concealed, generally speaking, unless this may in some instances be justifiable in order to sustain the courage and faith of the individual, and thus add to the chances of recovery.

His *co-operation* may be necessary, but co-operation without faith is usually hard to obtain. There may be a passive obedience to instructions, without that hearty entering in, which results from full-fledged confidence.

The physician more especially, would note the injurious effects of such a state of mind, in its retardation of the progress of recovery, and to his practice are these latter remarks more particularly applicable, though there is more of a necessity for "compatibility," between the Dental practitioner and his patients, than many are willing to admit. Not the kind of *surface* compatibility, with which the smooth-faced and oily-tongued swindler effects his object—though this is a gift, in itself a blessing, but made a curse by being perverted to bad uses—dishonesty, selfishness and deceit.

But to recur to the subject under consideration—there are times, when the probabilities are, that a certain course will entail suffering and loss: when the chances of success, are so absolutely obscure and remote, as to produce the $\frac{999}{1000}$ part of a certainty, that the result will be disastrous, and this too, when the interests of science and humanity do not warrant the running of the risk

When one knowingly, and persistently does so under such conditions, it may be said, that he has a decided inclination for running the risk.

The practice in vogue with some, of placing metallic fillings in contact with exposed pulps, is a case in point. This is often done. Were it not so, the cases of alveolar abscess, under treatment or otherwise, *known* to be the effect of such practice, would be materially lessened.

Suppose a mouth presents, with teeth to be examined. There is, or has been, trouble. A prominent front tooth for instance, having been filled, while in and under the above specified conditions, has become diseased, discolored and a source of offense—though the operation was pronounced by the dentist, or as a matter of course, expected by the recipient, to be "all right." Though the ill effects might not have been immediately apparent, the ultimate sure results, should have been foreseen: to withstand which, we have found that in such cases, and with such treatment, the recuperative and combative powers of nature were powerless.

At such times, it is clearly wrong to run the risk. There are times, when the pulp is left with only the protection which a thin layer of dentine affords, and when it is difficult to determine as to the safety of the operation—it being modified and varied, not only by the thickness of the septum but, by the present and future health of the individual. In such cases, a certain amount of risk is unavoidably run, if any attempt is made to save the tooth. But, where doubt exists, much, if not all of the danger, may usually be removed by proper precautions.

"An ounce of prevention is worth a pound of cure," especially a partial cure; for, "if a *tooth* die, shall it live again?"

In a case like the foregoing, a non-conductor, like the oxy-chloride of zinc, if placed over the frail covering of dentine, of sufficient thickness to prevent its disintegration, while filling over permanently *with care*, would usually preclude all trouble from thermal changes or pressure of the filling.

I am aware, that this treatment would be called in question by some—as it has been asserted, that a capping of the oxy-chloride of zinc, where but a thin layer, or floor of dentine, separates it from the pulp, will, owing to its caustic properties, cause congestion of that organ, by acting through the tooth substance—the result being more unfavorable than when it is placed in direct contact with the pulp itself. But the fact that, in only a single instance in my own practice, has it came to my knowledge of any trouble having arisen when this method has been adopted, is my warrant for continuing it.

Those who object to it, would not, probably, advocate the destruction of the pulp under such conditions, but the substitution of some substance with neutral, or unirritating properties.

A method of practice successful with some, might fail with others from some slight modifying cause. The injunction well comes in here, "make haste to make money slowly"—keep past and probable future results before the mind, and let caution guided by experience, have its perfect work.

An old, reprehensible, and half-way method of treating teeth with exposed pulps, though not so common as formerly, has not quite fallen into disuse. An aching tooth, is quieted by the destruction of the pulp. Where it dies, there it is buried. A filling in the crown, seals up the door of the sepulchre. The pulp decomposes, and its presence, in consequence of the foul and noxious gases emanating from it, proves a source of irritation, provoking periodontitis, and alveolar abscess. Evidence of this in two or more cases recently treated in this way, has been presented to my notice. This is another way of running the risk.

Next, a tooth is dead—which the patient may suspect or not, but as it has never made trouble, wishes it filled. The dentist cleans out the pulp and crown cavity, and without any other preliminary treatment, immediately fills one or both. Owing to the irritation produced, or because the pent up pus can find no exit, the gum swells, and much suffering is experienced, until nature finds another channel for its escape through the surrounding or adjacent tissues. Here was latent disease, requiring only a little irritation to develop the consequences, or it may have been a chronic, sluggish abscess, discharging through the canal of the root, and so have caused no inconvenience, excepting perhaps, being somewhat offensive.

How easy it is to ascertain the state of such a tooth, before inserting a permanent filling, it is needless to set forth—or what the method of treatment would be, in order to make it tolerable and harmless, were it found to be in the condition above described. The majority of teeth that have been dead any length of time, are more or less diseased, and it is always risky, as most are aware, to at once introduce a permanent filling.

Another patient calls for filling, and wishes good operations, with the best, most innocuous and permanent material. The cavities are large, difficult of access, and require much time, with thoroughness of preparation, to insure the greatest degree of success.

The operator, though aware that well inserted gold fillings are the best, and though he would usually advise such, feels at this time, very much like lightening his burdens. *Convenience*, is the arbiter. The carious teeth are back, the fillings will not be seen, amalgam is very good, and withal—cheaper, *he says.*

It is such a waste of nervous energy—he is so fatigued—time is limited—if she makes an engagement for another sitting, she may not keep it, *he thinks.* So he is tempted to sacrifice the *ought* to the *ought not*—to *Convenience.*

On a par with this, or below it, is the practice of selecting and filling the simple and small cavities, and passing by the large and difficult ones, without notifying the patient of the presence of the latter, or under the pretense that they are "too far gone to pay for filling."

In running the risk, in such or similar cases, the springs of action are varied. It might not be charitable to assert, that they are worked by an I-don't-care-if-I-get-my-money feeling, or a lack of ability, or inclination to do well, "whatever the hand findeth to do," by carelessness, indolence, or a lack of that patience, perseverance, or discrimination, which will not allow one to stop short of the essential final touch, call an unfinished operation finished, and let it pass, lest some waiting now-or-never patient elude his grasp. It is not reasonable or charitable to suppose, that he would wish to "redeem the time," in order to make every moment tell—for money—or do injustice to a "bird in hand," rather than lose the chance of capturing one or "two in the bush," or that in order to divert golden streams from other channels into his pockets, he would be willing to betray the confidence reposed in his faithfulness and honesty!

Of most, "we are persuaded better things." But, whatever may be the influences, or motives reaching back of all action, the first thing usually scanned by human eye is the result, though motives, and results are, at times, so equally apparent that neither can be disguised.

But at times, the interests of science and humanity, fully warrant the risk, attendant upon experimentative treatment, though rarely, if ever, to such an extent as to endanger human existence, excepting a foregone conclusion has been established by sufficient evidence, that without it, the case would be hopeless.

Circumstances might be such even, as to render it necessary to run the risk of sacrificing one life in order to save a greater number—even if such a termination were certain—so that an evil though it were, of no small magnitude, it might become right or necessary to permit it, in order to avert an evil, or aggregation of evils of a more serious nature. What may seem an evil to the finite mind, may be permitted by God, to bring about results, which render the sum total of providences an inestimable blessing.

If, in the Providence of God it becomes necessary to carry out this principle, when momentous issues are involved, thus justifying in some instances, what may seem extreme measures, it certainly would be just to apply it in things of minor importance. And, thus it is, though in a lesser degree, that this principle has to be adopted in the attempted salvation of the pulps of teeth. Here, a certain amount of risk has to be run. In what cases, and under what conditions it *should* be, is a matter of difference of opinion. But the allusion is here made to the practice most commonly and successfully in vogue of what is, or should be, the delicate, careful and skillful application to exposed pulps of the oxy-chloride of zinc capping.

Some, would include under such treatment, only the freshly exposed and healthy pulps.

Others, would consider as proper for such, all those possessed of *any* vitality. "Let every man be fully persuaded in his own mind" after sifting and balancing results, failures and successes, and *causes* of failures and successes in his own, and others practice.

Here is a legitimate and proper field for running the risk; but not heedlessly, extravagantly or blindly, or with mere mechanical dexterity, without regard to conditions, or consequences. The great and positive advantage, resulting from the salvation of a goodly number of exposed pulps, overbalances the not irremediable evils, resulting from a lesser number of failures. Here the interests of dental science are subserved, and running the risk is promotive of both present and future good, notwithstanding the sometimes attendant evils.

The old method of destroying the pulp, and filling from apex to crown, was not and is not entirely without present and prospective risk, and there is hardly a class of operations in our specialty unattended therewith.

It is plain that *leaving alone* is sometimes more worthy of censure than an attempt to remedy. A case of alveolar abscess, for instance, should not be left to produce its injurious effects. In allowing it to remain without treatment, when our advice to the contrary would be heeded, is in most instances wrong, for it is running the risk of serious consequences, and if not prepared for the work, is it not our duty to become so, or recommend the patient to go elsewhere.

While the omission of duty is culpable, the selfish, heedless, or blundering commission of wrong acts may be so to a greater extent, especially where permanent injury is inflicted upon an individual, by taking advantage of his ignorance, or even willfulness.

If natural, sound and serviceable, but ungainly and malpositioned teeth, are, by the request or assent of the patient, or otherwise, extracted, merely because they do not present the most beautiful appearance desirable, the operator might run the risk, with all his skill, of not inserting an equally valuable denture, and it is questionable, to say the least, whether such risk ought to be run, and whether such a dentist is not verily guilty. Many cases came under notice, in which the unfortunate patients would give much, could the lost, homely, but serviceable natural teeth be restored.

Mrs. A., with an extremely irregular, but sound set of under-teeth, with an aggregation of grinding surfaces singularly uneven, with an excessive lingual deflection of the left bicuspids, with a deviation of from one to three and one-half lines from a level of the combined grinding surfaces of the left double teeth, that of the first molar presenting the lowest, with an up-hill arrangement of the second molars of each side, with stubby, projecting front teeth, etc., and with which it is almost impossible to antagonize artificial ones with any prospect of giving anything more than very poor satisfaction, but with the upper natural side and back teeth, somehow so arranged and antagonized therewith, as to operate without much, if any, inconvenience—chances to want a few upper front teeth removed, and artificial ones inserted. But the dentist advises, and convinces the patient sufficiently to obtain her acquiescence, that a full upper set had better be inserted, and removes not only the decayed incisors, but the numerous sound posterior teeth.

The risk to be incurred in such a case of giving anything approaching satisfaction, to say nothing of the robbery itself, would seemingly, be sufficiently apparent to deter any dentist from assuming such a needless responsibility. Not so, however. Consequences—an ever abiding sense with the patient, of an inflicted wrong, and an ever present inconvenience and want, resulting from the loss of organs, whose efficiency and value can never be equaled by any artificial appliance, with the sacrifice of comfort, and perhaps of health.

This exceptional case illustrates a principle. Paltry considerations of profit, is the mainspring which usually impels to such sacrifices, so far as the dentist is concerned in making them, and this is the motive which too frequently governs in many similar cases.

But can not one reap without sowing tares? He can, if there is no lack of those aspirations which tend to elevate, consecrate and render noble, every act, and every calling, upon, and in connection with which, he can legitimately "ask, seek and find" God's blessing. True, the harvest may, in many instances, be large, though wrongfully secured, for tares produce abundantly, but it shall only be golden in the highest sense, if the sheaves which are gathered into the garner, bear "not corruptible things as silver and gold," but those of a more enduring nature.

